# Using Fitness Surfaces to Better Link Conservation Breeding Programmes With Wild Population Recovery

**DOI:** 10.1111/mec.17798

**Published:** 2025-05-15

**Authors:** Drew Sauve, Hana Thompson, Amy A. Chabot, Denis Réale

**Affiliations:** ^1^ Department of Research and Conservation African Lion Safari Ontario Canada; ^2^ Département Des Sciences Biologiques Université du Québec à Montréal Québec Canada

**Keywords:** captive populations, conservation genetics, contemporary evolution, phenotypic plasticity, quantitative genetics, wildlife management

## Abstract

Fitness surfaces offer a valuable tool for bridging the gap between captive breeding programmes and wild populations. By quantifying the relationship between phenotypes and reproductive success in captive and wild settings, fitness surfaces can help identify the fitness consequences of phenotypic change in either environment. Measuring fitness surfaces in captive and wild populations from the same species would help us to predict the success of reintroduction efforts and help inform the selection of release candidates. Overall, the inclusion of fitness surface estimates into conservation breeding programmes increases the effectiveness of reintroduction efforts and should improve our understanding of evolution at the interface of human‐managed and wild populations. Beyond conservation breeding, fitness surfaces may have applications for at‐risk species such as predicting outcomes in range expansions, translocation or under changing environmental conditions.

## 1 Introduction

Anthropogenic activities alter the environments that most species experience, and these changing environments are expected to alter selection pressures (Gienapp et al. 2014; Otto 2018; Charmantier et al. 2024; Edelsparre et al. 2024). Shifting environments present a challenge for conservationists. While we may eventually be able to predict whether wild populations will adapt to rapid environmental change, our ability to intervene and assist adaptation remains limited (Bowgen et al. 2022). Conservation breeding programmes might offer a unique opportunity to bridge this gap. By managing captive environments, we can potentially shift selection pressures towards those experienced in the wild. Additionally, we need to be able to identify captive individuals with a greater likelihood of success upon release. However, to effectively implement conservation breeding programmes, we must improve our empirical understanding of fitness and selection in both captive and wild populations (Crates et al. 2022; Frankham 2008; Frankham et al. 1986; Gilligan and Frankham 2003; Sauve et al. 2022).

Integrating fitness surfaces into conservation breeding and management of populations intended for release could allow us to better support wild populations and address challenges faced by conservation breeding programmes. The challenges around predicting adaptation, managing selection in captivity and intentionally releasing specific phenotypes are undeniably difficult. However, fitness surfaces, which have deep roots in evolutionary biology (Wright 1932), would allow us to quantify the relationship between traits and fitness in both captive and wild settings. Here we use the term fitness surface to describe the function that predicts an individual's fitness within an environment given its phenotypic value (Lande 1976; Arnold 2003; Morrissey and Sakrejda 2013; Walsh and Lynch 2018). Following this definition, we note that the fitness surface is distinct from the fitness landscape or adaptive landscape. Fitness landscapes or adaptive landscapes examine how the mean fitness of a population is expected to change given variation in parameters (e.g., mean and variance for a normally distributed trait) of the population distribution of phenotypes (Simpson 1944, 1953; Walsh and Lynch 2018).

**FIGURE 1:**
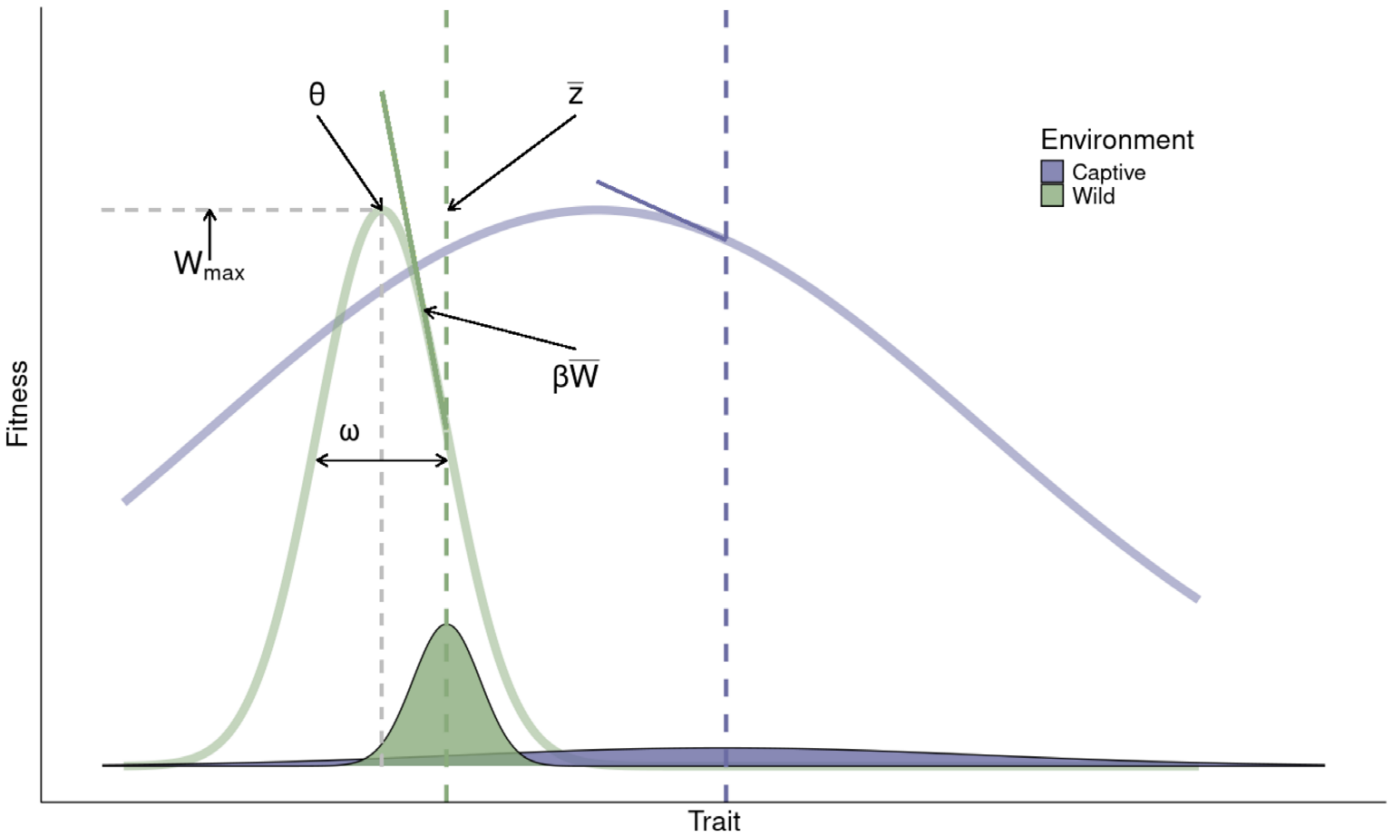
Theoretical scenario of two fitness functions and two phenotypic distributions for the same trait in a captive and a wild population. In this case, the average trait value and variance is smaller in the wild than in the captive environment. Within each environment, the fitness optimum (θ) is at a smaller value compared to the average phenotype, leading to possible evolution of the populations towards a smaller range of trait values. The fitness optimum (θ), maximum fitness (Wmax), average trait value ($\bar{z}$), width of the fitness function (ω) and strength of selection (β $\bar{W}$) are all indicated for the wild population.

Fitness surfaces have seen limited empirical measurement and application in conservation biology (for a few examples in wild populations see Blows et al. 2003; Garant et al. 2007; Beausoleil et al. 2023), but provide a means to link phenotypes and fitness, and predict adaptive divergence between environments (Arnold 2023; Arnold et al. 2001; Beausoleil et al. 2023). In the context of the conservation breeding environment compared to a release (wild) environment, fitness surfaces can help inform whether population augmentation is causing divergence from wild type, which phenotypes should have high fitness in the release environment, and the relative roles of plasticity and selection in novel conditions. These insights in turn would improve our ability to use conservation breeding to support wild populations, improve our theoretical models of captive selection and its consequences, and assist us in predicting the impact of human induced environmental change on wild populations (Crates et al. 2022; Lynch and O'Hely 2001; McPhee and McPhee 2012; Pelletier et al. 2009; Schulte‐Hostedde and Mastromonaco 2015). To illustrate the potential for fitness surfaces, we can imagine a simple surface for one trait using the fitness function:

$$W\left(z\right)=\text{Wmax}∙\exp\left(\frac{-{\left(z-\theta \right)}^{2}}{2\omega^{2}}\right)$$

where fitness (W) is a function of a trait z. This Gaussian function illustrates stabilising selection, with a maximum fitness value of Wmax, an optimal trait value of θ and the width of the fitness function ω. Two different Gaussian fitness functions describing the same trait and fitness can characterise the captive versus wild populations (Figure 1), each with their own trait optimum, width and maximum fitness. In this example, we imagine that the fitness function for a population of the species held in captivity is broader (larger ω value) when compared to a fitness function for the same species in the wild (Figure 1), but it could be narrower. In this scenario, the broader fitness function in captivity might be due to relaxed selection in the captive environment (e.g., McPhee and McPhee 2012). Further, we show two tangent lines indicating the rate of change in fitness according to the fitness function at the point of the average trait value (Figure 1). In reality, an estimated fitness surface could be “rugged” with the possibility of multiple fitness peaks in each environment (e.g., Beausoleil et al. 2023). Given the assumed relative stability of conditions in a managed environment, we might anticipate a smoother fitness surface with fewer valleys in captivity due to relaxed selection pressures. Importantly, a practitioner's goal will affect their approach for estimating the fitness surface. For the goal of predicting fitness from an individual phenotype, a spline approach that captures the complexities in the shape of a fitness surface might be most suitable (Martin and Gould 2020; Schluter 1988). Alternatively, inferences about adaptation might be more easily made by estimating the parameters of a multiple regression (Lande and Arnold 1983). Either way, it would be logical to first estimate the fitness surface with a flexible spline approach to help inform the function that should be used to estimate selection (Schluter 1988; Schluter and Nychka 1994).

Herein, we describe how fitness surfaces measured in wild and captive environments will help measure the potential impacts of selection and plasticity on reintroduced individuals. We then discuss how detailed fitness surfaces could guide the selection of release candidates.

## 2 Potential Uses of Fitness Surfaces

In our view, the parameters of greatest interest to breeding programme managers and conservationists participating in augmentations or reintroductions will be the optimal trait value (θ) and the strength of selection acting on a trait in each environment (the derivative of the fitness function evaluated at the average trait value for each population). From these parameters, we can determine how and if selection is shaping phenotypes in the wild and captivity (Section 2.1) and thus predict which phenotypes are likely to have high fitness in the wild (Section 2.2).

### 2.1 Observing Phenotypic Change Across the Fitness Surface

To determine whether phenotypic selection (i.e., the covariance between phenotypic traits and fitness) will lead to evolutionary change in a given environment, we must assess the heritability of the trait in question. Narrow‐sense heritability quantifies the proportion of phenotypic variance attributable to additive genetic variance, in other words, the extent to which offspring resemble their parents due to shared genetic contributions (Falconer and Mackay 1996; Lynch and Walsh 1998).

**FIGURE 2:**
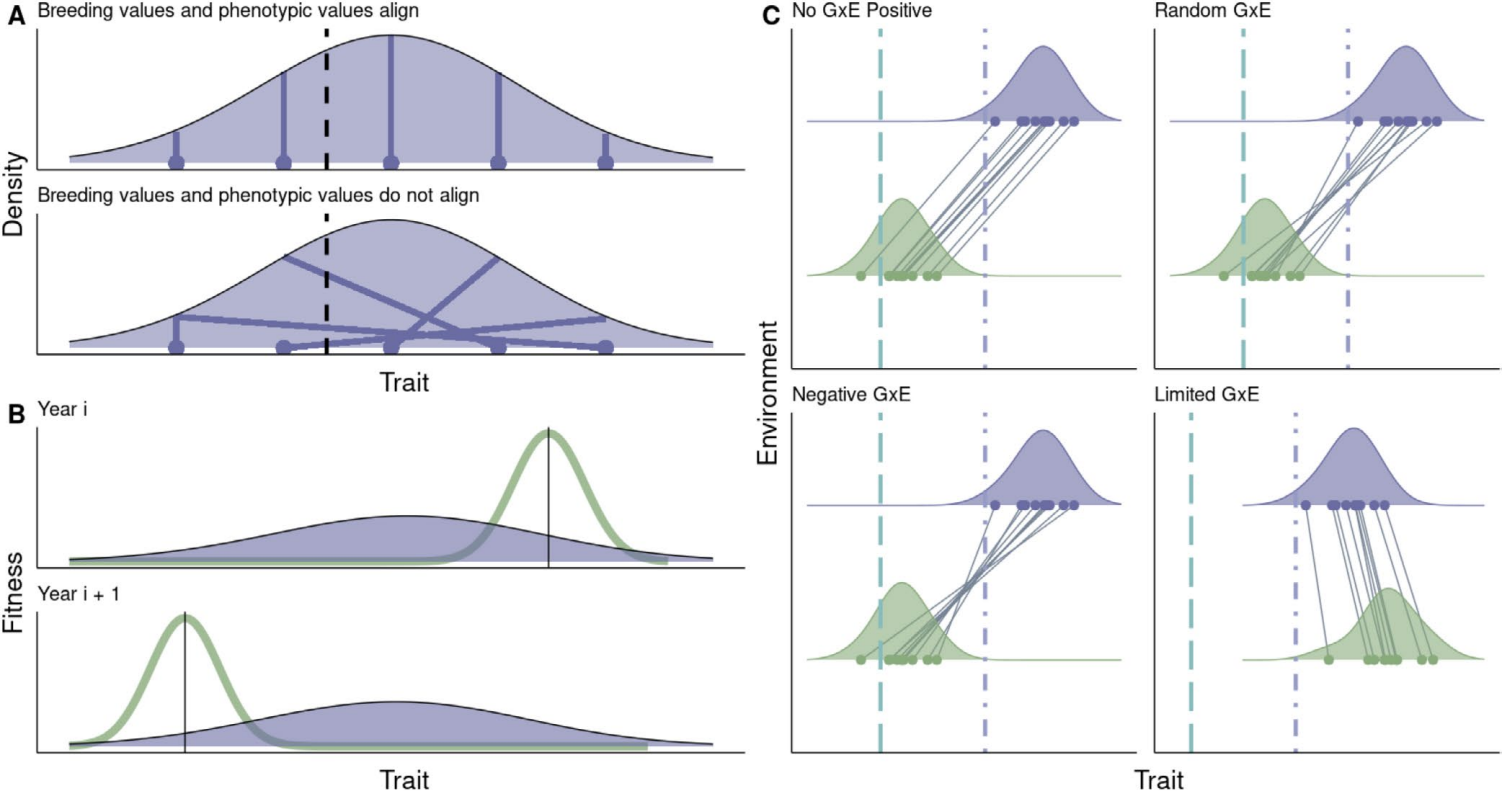
(A) A hypothetical relationship between breeding values and phenotypic values. The arbitrary density indicates the range of phenotypic values observed in captivity and the vertical dashed black line indicates a fitness optimum. Phenotypic values closer to the optimum are expected to have higher fitness values than those further away from this optimum (as determined by a fitness function). In the case that there is limited plasticity or there is limited environmental variation, phenotypic values are a good predictor of breeding values (top panel). Therefore, selection (which operates on phenotypic values) will result in a between‐generation change in trait values (adaptation by natural selection). However, it is also possible that breeding values do not align well with phenotypic values (bottom panel) because the environment that genotypes experience is heterogeneous and genotypes plastically respond to this variation (e.g., some facilities provide different diets or healthcare). In this case, selection acting on phenotypic values will result in a within generation change of phenotypes, but not a between generation change. (B) Two years of study in which the fitness function in the wild is known and can potentially be used to inform the choice of released individuals. In each year, the light green open function is the fitness function in the wild context and the dark purple distribution is the distribution of phenotypic values in captivity. In a simple scenario in which there is limited plastic response to the release (wild) environment, managers could aim to release phenotypes that are expected to have a higher fitness value according to their knowledge of the wild fitness function. (C) Hypothetical genotype by environment interactions that are possible across the captive and wild environments. In all scenarios the purple upper distribution corresponds to phenotypic or breeding values in the captive environment, while the green bottom distribution correspond to phenotypic or breeding values in the wild environment. The far left long‐dash teal vertical line corresponds to the fitness optimum in the wild, while the right dash‐dot purple vertical line corresponds to the fitness optimum in captivity. Unbroken diagonal lines connect the phenotype expressed by an individual or the breeding value expressed by a genotype in the captive and wild environments. There could be a positive phenotypic or genetic cross‐environment correlation with limited or no GxE in which the largest phenotypic or breeding values in captivity correspond to the largest phenotypic or breeding values in the wild (top left). No cross‐environment phenotypic or genetic correlation in which the phenotypic or breeding value in one context does not predict the relative value in the other (top right). A negative cross‐environment phenotypic or genetic correlation where the largest and smallest phenotypic or breeding values correspond to the smallest and largest values in the wild respectively. Finally, the captive environment could strongly alter the phenotypic or breeding values expressed in the wild, resulting in phenotypes that are distant from the wild fitness optimum.

If a trait has a high heritability in a specific environment, then phenotypic selection is likely to reflect underlying genetic selection, meaning the covariance between phenotype and fitness is matched by a covariance between breeding values and fitness. Breeding values represent the additive genetic contribution of an individual relative to the population mean, or the sum of the average effects of alleles influencing the trait (Falconer and Mackay 1996; Lynch and Walsh 1998). In this sense, heritability determines how well genetic differences (breeding values) are expressed in phenotypes and passed to the next generation. For example, if body size is a heritable trait, and larger individuals consistently survive or reproduce more, selection will increase the average body size in the next generation and shift allele frequencies accordingly (Figure 2A, top panel). However, if phenotypic variation is largely due to environmental factors (most of the observation variation is the result of plastic responses to environmental heterogeneity), heritability will be low. In such cases, breeding values may not reliably predict phenotypic values (Figure 2A, bottom panel), and phenotypic selection may not translate into evolutionary change (Price et al. 1988; Rausher 1991).

Further, the evolvability of specific traits could help guide which traits should be monitored in conservation breeding programmes. If a trait is not expected to evolve, it may be less critical to monitor. Evolvability, which refers to the expected response to selection per unit of selection, provides a quantitative way to assess this potential (Houle 1992). Traits with a higher evolvability are expected to have a higher potential response to selection. For multiple traits, trait combinations with the most genetic variance combined with expected or simulated selection can allow researchers to assess multivariate evolvability across fitness landscapes (Schluter 1996; Hansen and Houle 2008; Walsh and Blows 2009; Chenoweth et al. 2010). Managers could use evolvability estimates in combination with a fitness surface to prioritise monitoring and management of traits that are responsive to selection and likely to affect reproductive success or survival. In our view, traits that may be most important for conservation managers to monitor would be traits with high evolvability that impact survival and/or reproduction.

Most importantly, examining trait changes in the context of wild and captive fitness surfaces will help us understand potential fitness consequences of any phenotypic changes. To understand how phenotypes change in captivity, we can examine how average phenotypic and breeding values change over time/generations or move across the captive environment's fitness surface. For example, studies of conservation breeding programmes of Houbara bustards ( *Chlamydotis undulata* ) and eastern loggerhead shrikes ( *Lanius ludovicianus migrans* ) have identified phenotypic and genetic changes over time, but we need to know, as a next step, whether these changes are likely to affect fitness in captivity or the wild (Chargé et al. 2014; Sauve et al. 2025). Fitness surfaces measured in each environment provide a framework through which we can make these predictions. Importantly, phenotypic change within either environment could make reintroduction more difficult if average trait values move in a direction that results in wild‐expressed phenotypes being further away from a fitness optimum. Identifying the contributor(s) to these phenotypic changes may help managers to avoid undesirable change. Various empirical studies and reviews outline how to use animal models to try to disentangle and estimate the contributions of selection, genetic drift, gene flow, environmental change and demographic change to observed phenotypic changes (Bonnet et al. 2019; Hadfield et al. 2010; Reid et al. 2021; Sauve et al. 2022; Wolak and Reid 2017). Nonadditive genetic effects (e.g., dominance and epistasis) may also play a role in shaping phenotype and fitness in small populations (Wolak and Keller 2014). Non‐additive genetic effects could potentially complicate interpretations of fitness surfaces and may be worth further investigation in many of the small populations that conservation biologists are often working with in captivity or the wild.

### 2.2 Identifying Ideal Individuals for Release

If we can predict an individual's phenotypic expression upon release (see Section 3.2), understanding the fitness function dynamics in the wild will allow us to identify candidate individuals for release in cases of population augmentation (Section 2.1.2). Studies of wild long‐term populations have begun to identify how fitness functions and selection vary over time and space and what the environmental drivers of these functions might be (Bonnet and Postma 2018; de Villemereuil et al. 2020; Sauve et al. 2024). Understanding how these functions vary and change is necessary to make predictions about individual fitness upon release or future adaptation. Assuming we know how a phenotype will be expressed upon release, we could choose individuals for release that are closer to that year's fitness optimum (we use year, but any ecologically relevant time step could be considered; Figure 2B. Box 1). This approach will be most straightforward to implement if the fitness function and predicted optimal phenotype are consistent between years. If the fitness function changes from year to year, it may be important to vary which phenotypes are chosen for release each year (Figure 2B). This could be difficult to implement in practice if there is a lot of fluctuation in the strength and/or direction of selection from year to year. In these cases, it will be important to understand whether and to what extent selection from year to year is autocorrelated (Chevin et al. 2015). If selection fluctuates between years, but is predictably autocorrelated, predictions about the optimal phenotype could still be made based on information from the previous year. Alternatively, it may be wise to consider a diversifying bet‐hedging strategy (i.e., not putting all your eggs in one basket), releasing a diversity of phenotypes to ensure that all known or possible phenotypic optima are released (Philippi and Seger 1989; Slatkin 1974). Fitness surfaces can also be generated separately for males and females for species that are sexually dimorphic, in which optimal phenotypes may be quite different between sexes (e.g., orange colouration is an important trait for male, but not female, fitness in guppies; Houde 1987). Fitness surfaces offer a flexible approach that can be adjusted based on species traits and environmental data and informed by previous releases.

Thus far, we have largely focused on a case where there is one wild population that is the target of reintroductions, and one captive population that is the source for reintroductions. However, in many programmes there may be multiple reintroduction sites and/or multiple source populations used to initiate the captive breeding population. If the populations of interest differ in some way (e.g., size, environment, extinction risk, genetic differentiation), this can lead to debates about the potential benefits of genetic rescue to increase genetic diversity with the potential risk of outbreeding depression or introducing maladaptive variation into locally adapted populations (Carlson et al. 2014; Edmands 2007; Frankham et al. 2011; Ralls et al. 2020). For conservation practitioners, this debate can make it difficult to determine which course of action is best for their species and may ultimately impede conservation efforts (Ralls et al. 2018). There already exist guidelines for determining when genetic rescue is appropriate, based on theoretical and empirical work (e.g., Frankham 2015; Hoffmann et al. 2021; Ralls et al. 2018). We argue that fitness surfaces could be particularly helpful alongside these guidelines, in combination with demographic, life history, and/or molecular data, where available. Consider a scenario in which a breeding programme has been initiated from two wild populations, and reintroductions are planned for both populations. Conservation managers may be unsure whether to breed all individuals or keep them separated by origin population, as well as whether to reintroduce individuals from population A to population B, and vice versa. There are several possibilities for incorporating fitness surfaces depending on what other data are available. For example, populations might be differentiated in terms of phenotype, additive genetic variance, or molecular markers. However, relying solely on one measure of differentiation (e.g., F_ST_) may underestimate meaningful differences in quantitative traits that reflect local adaptation. Even when molecular differentiation is low, as seen in Corsican blue tits ( *Cyanistes caeruleus* ) with values of 0.006–0.008 (Perrier et al. 2018), common garden experiments have revealed genetic divergence in quantitative traits. In cases of genetic or phenotypic differentiation, managers might be weighing the potential benefit of combining the populations in the breeding programme to increase genetic diversity, with the potential risk of outbreeding depression and the introduction of deleterious variation. Generating fitness surfaces could allow conservation managers to compare fitness optima and predict the fitness of a given phenotype in both environments. If the two populations have similar fitness optima, and interbreeding maintains the optimum phenotype, then interbreeding between populations may be an appropriate approach. For example, fitness was maintained when stickleback under similar ecological conditions (benthic vs. limnetic) interbred, even when they were from different lakes (Rundle et al. 2000). Alternatively, if the fitness optima are very different, the conservation programme may conclude that the genetic differentiation represents adaptive differences that should be maintained. However, in this case, fitness surfaces may offer the best of both worlds—enabling the combination of populations in the breeding programme to increase genetic diversity, while providing insight into which phenotypes are best suited for release based on their proximity to the fitness optima of each population.

Finally, the measure of fitness used to generate a fitness surface will likely matter. If managers know which vital rates are impacting demography, they may want to focus on using fitness surfaces that are generated using demographic parameters that are expected to improve demography. For example, following a translocation of desert tortoises ( *Gopherus agassizii* ) introduced individuals had similar survival to residents but much lower fecundity (Mulder et al. 2017). In this case, optimising phenotypes for survival without considering fecundity is unlikely to improve the demographic situation. More broadly, fitness surfaces that incorporate multiple components of fitness or that focus on the components most limiting population growth may provide more useful guidance for management (de Kroon et al. 1986).

## 3 Individual by Environment and Genotype by Environment Interactions

A key insight that the combined captive‐wild fitness surface can provide is predicting how individuals and their offspring will reproduce and survive once released (Section 3.1). To predict survival and reproduction, we need to know how some fitness‐related traits may change in the release environment because of differences between the release and captive environment (diet, interspecific interaction, etc.). Plastic changes may be influenced by environmental and genetic differences among individuals (individual by environment interactions or IxE). It's important to note that this refers to phenotypic traits that are flexible and can be altered by environmental factors, excluding those that are largely fixed by genetics or become immutable during development. However, we could use relatedness information (derived from pedigrees or molecular data) to determine if family groups exhibit similar responses to the release environment and ask whether variation in responses exists among family groups (genotype by environment interactions or GxE). Understanding the cross‐environment genetic correlation will be particularly important, as the presence, absence, strength and direction of these correlations will impact our ability to predict the phenotype of an individual's relatives upon release. Beyond flexible traits that vary with environmental conditions within an organism's lifespan, it will also be important to examine how captivity influences trait development (Section 3.2). Some traits, like phenological ones, can be quite flexible after development, while others may only be receptive to cues during specific developmental windows (West‐Eberhard 2003; Sultan 2017; Snell‐Rood et al. 2018). Additionally, the degree to which conditions vary across breeding facilities must be evaluated as environmental heterogeneity among facilities has the potential to impact both fitness surfaces and plastic responses (Section 3.3).

### 3.1 Some Examples of Plastic Responses to the Release Environment

Measuring plastic responses to the release environment is crucial because it will determine to what extent phenotypes and fitness can be predicted after release. Ideally, plastic responses will result in phenotypes post‐release that are closer to the phenotypic optimum in the wild (Figure 2C, top left, top right, & bottom left). For example, we can imagine that, on average, released individuals learn to reduce movement around predators. In this hypothetical scenario, conservation practitioners have implemented a predator training programme for the animals to encourage this predator avoidance behaviour. Keepers introduced a predator cue and recorded the movement activity of animals in the captive environment while wildlife ecologists recorded similar data on released animals in the wild. Ideally, we will observe an adaptive plastic response to the wild environment in which all released animals will adjust their phenotype to approach the wild fitness optimum (Figure 2C, top left, top right, bottom left, but not bottom right). In our hypothetical scenario, this would correspond to all animals decreasing their movement on average in the presence of predators compared to the captive environment. If there is limited variation in individual or genotype responses (limited IxE or GxE), but a positive cross‐environmental correlation between phenotypes or genotypes, individuals with the lowest or highest activity values in the captive environment also have the lowest or highest activity values in the release environment (Figure 2C, top left). Alternatively, there might be variation in responses to the wild environment (IxE and GxE), but no cross‐environmental correlation between captive phenotype and wild phenotype (Figure 2C, top right). In this case, we would be unable to predict an individual's phenotype in the wild based on captive measurements, but we might be able to preferentially release or breed family groups with a known response to the wild environment. However, we would have to critically evaluate the value of artificially selecting specific responses. At the other extreme, individuals might on average reduce movement in the wild, but individuals with the highest activity levels in captivity might have the lowest activity levels in the wild (Figure 2C, bottom left). In this scenario, we can still predict the release phenotype (although it might be counter intuitive) and therefore identify candidates for release. It will be important to try to determine if cross‐environment correlations (at the individual or genetic level) are common as they have the potential to impact decisions related to the release. Finally, individuals might not adjust their phenotypes much when released, either because of evolution in captivity and/or environmental conditions experienced in captivity (Figure 2C, bottom right). The repercussions of a lack of response will depend on the difference between the fitness functions in the wild and captivity, but are worth investigating to determine when individuals or family groups should be preferentially released based on their phenotype in the captive environment.

### 3.2 Multivariate Fitness Surfaces

**FIGURE 3:**
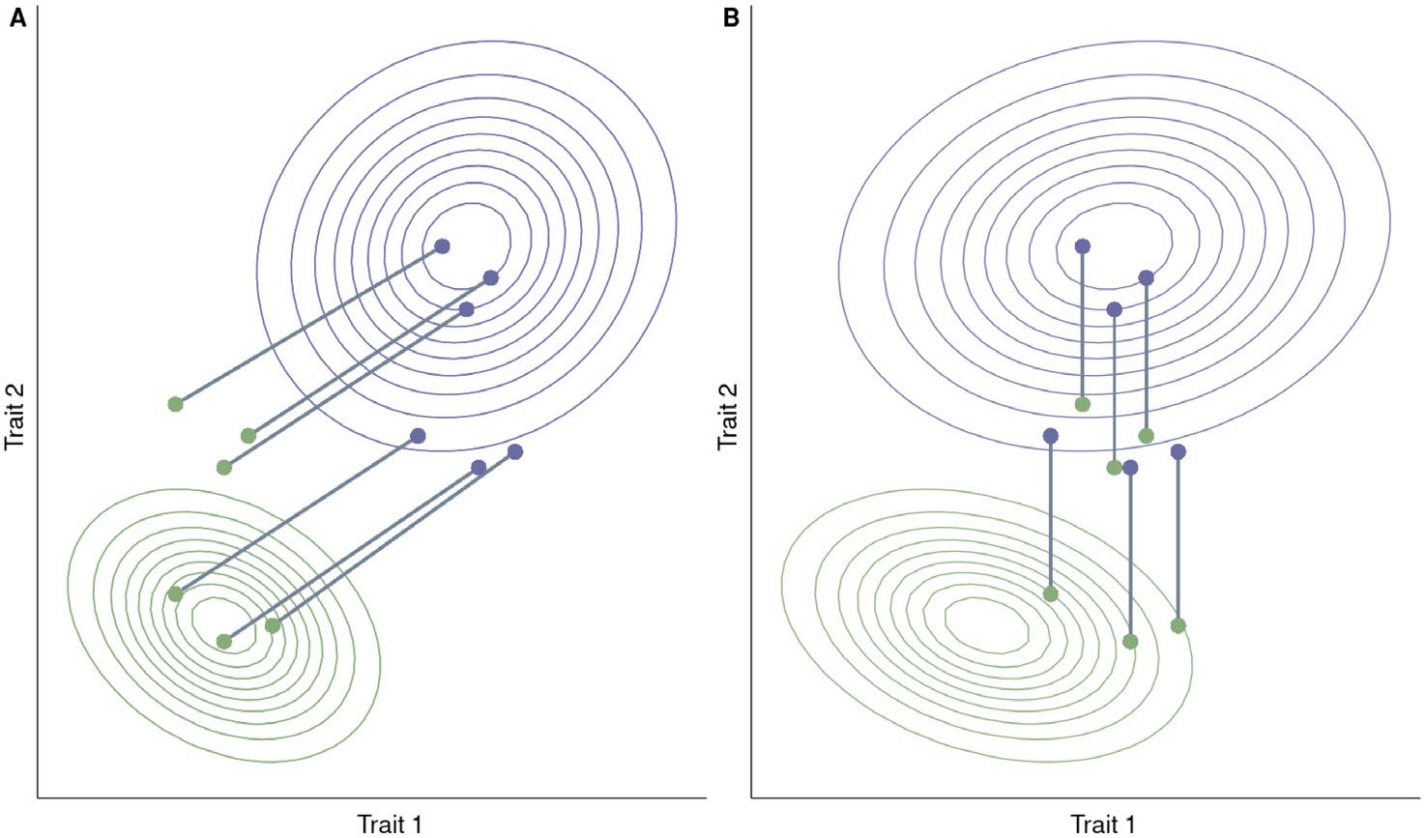
Fitness surfaces of the captive (purple) and wild (green) environments. Contours indicate the expected fitness of the multivariate phenotype consisting of two traits. The top right purple contours in both panels indicate a hypothetical fitness function for captive phenotypes while the bottom left green contours in both panels indicate a hypothetical fitness function for wild phenotypes. Purple points indicate phenotypes expressed by an individual or genotype in the captive environment, while green points indicate the phenotype expressed by an individual or genotype in the wild environment. Lines connecting purple and green points connect the phenotypes that the same individual/genotype expresses in each environment. (A) If phenotypes are optimised using only measurements on trait 1 they may not have all the information needed to accurately predict fitness outcomes upon release. (B) Some traits may plastically response to the wild environment, while others may be permanently altered. Here individuals/genotypes are plastically adjusting trait 2 in the release environment, while trait 1 remains the same across both.

So far, we have expressed our ideas in a univariate framework, but selection in the wild and in conservation breeding programmes will likely operate on many aspects of an organism. To predict fitness from phenotypes, we have to consider the multiple traits likely contributing to fitness variation (Phillips and Arnold 1989; Schluter 1988). Neglecting to consider multiple traits could mean that attempted predictions of fitness upon release will be inaccurate. For example, we might imagine that we are trying to use fitness surfaces to predict fitness in a release programme, but neglect to investigate a secondary trait that also contributes to variation in fitness. We might have studied this primary trait and understood that there is a plastic response to the wild environment, such that individuals or genotypes express a smaller phenotypic value for trait 1 that is closer to the phenotypic optimum when released (Figure 3A). However, because we neglected to measure this second trait, half of our released individuals or genotypes perform poorly when they express values for a second trait that are not near the wild fitness optimum (Figure 3A). While multivariate approaches could improve predictive power, it remains challenging to ensure that all fitness‐relevant traits have been identified and measured, and we caution that unmeasured traits could still compromise the accuracy of fitness predictions upon release.

It is well noted that to predict phenotypic or evolutionary trait change caused by selection, we will also need to account for direct selection, indirect selection and genetic constraints (Houle 1991; Lande and Arnold 1983; Phillips and Arnold 1989). Phenotypic and genetic correlations mean that the within and between generation responses of traits to selection will not be independent. Alignment of trait correlations and selection can lead to strong responses to selection, while antagonistic correlations will result in constrained responses. For example, there are some suggestions that selection for tameness in animals results in a common suite of trait changes (Belyaev 1979; but see Lord et al. 2020).

### 3.3 Developmental and Transgenerational Effects

**FIGURE 4:**
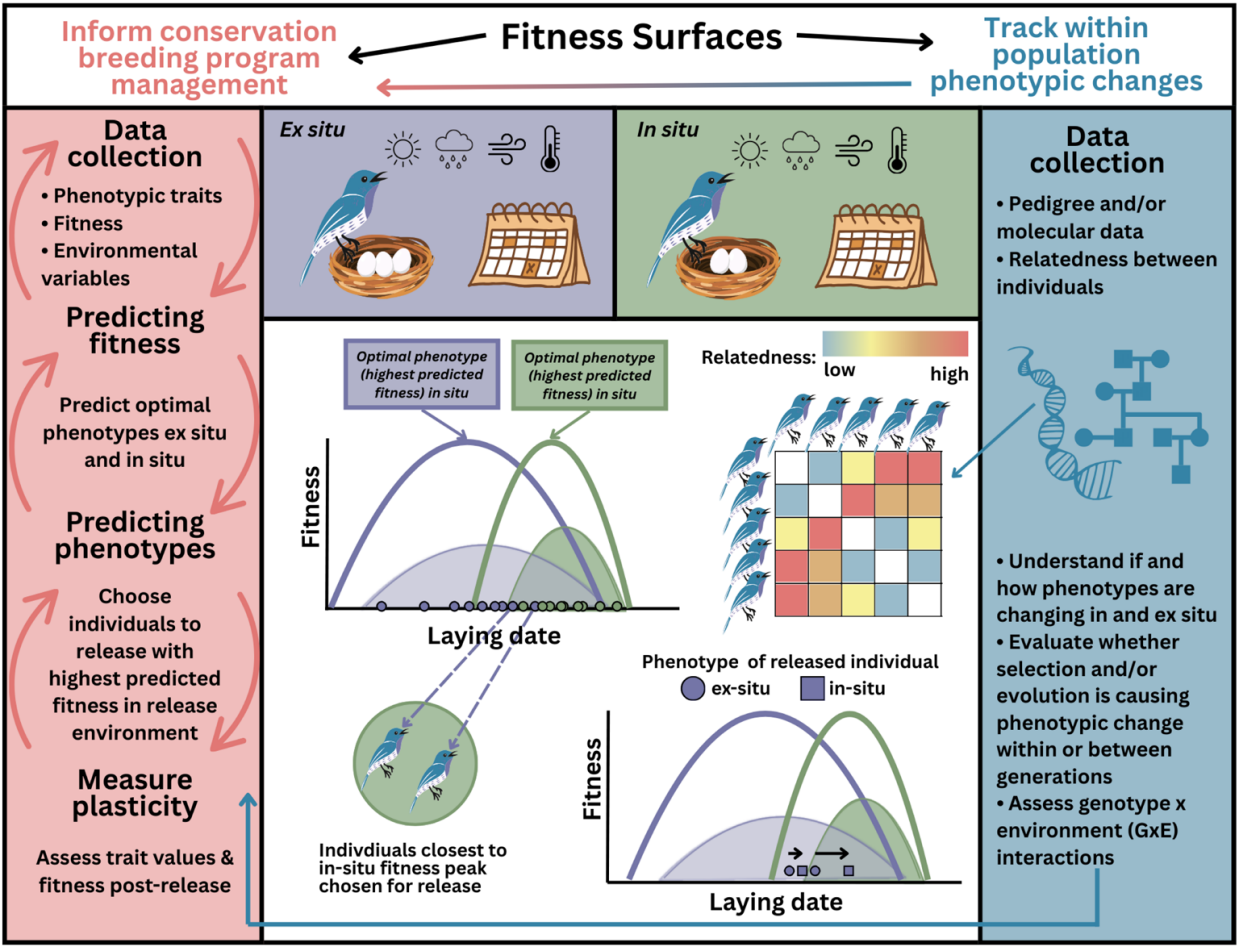
Fitness surfaces to inform conservation breeding programme management and track within‐population change. Top panels: Conservation managers could collect the same data in the captive (purple) and wild (green) populations traits of interest (e.g., laying date), fitness (e.g., number of eggs), and environmental variables. This data can then be used to generate fitness surfaces for both environments (middle, top left). The solid purple and green lines show the predicted fitness for a given phenotype (in this case, laying date), and the shaded distributions show the phenotypic distribution of laying date in both environments. Points along the x‐axis represent individual values. From these data, we could then identify individuals that are predicted to express phenotypes closest to the fitness peak in the release environment (middle, bottom left). After release, conservation managers can assess trait values and fitness post‐release and determine if, and to what extent, individuals are responding plastically to their environment. Fitness surfaces can also be used to track within population phenotypic changes (right panel). When relatedness between individuals is included (using either pedigrees or molecular data), we can assess the heritability of a given trait and evaluate if and to what extent evolution is causing phenotypic change between generations in each environment (see Section 2.1). This information can then further inform breeding programme management.

Some traits are flexibly adjusted by individuals throughout their lifetime. For example, some individuals in populations of partially migratory birds will migrate in a given year, but remain on the breeding grounds in another (Buchan et al. 2019; Chapman et al. 2011). However, some plastic responses occur in response to environmental cues early in development, the effects of which may or may not be reversible (West‐Eberhard 2003). Similarly, some traits might plastically respond to the release environment, while others will be canalised during development in captivity (Figure 3B). This has important implications for conservation breeding programmes as the environment an individual experiences early in their development could be very different from the environment they will experience if released into the wild. For example, many reptile species change colouration in response to the environment for a variety of purposes (e.g., camouflage, thermal regulation; Cooper and Greenberg 1992), such as captive‐raised Blanding's turtle ( *Emydoidea blandingii* ) hatchlings which plastically respond to the colour of the housing in which they are raised: turtles in black tubs exhibit darker colouration than those held in white tubs (S. J. Stanger Guy, personal communication; Rowe et al. 2017). If this response is adaptive in the wild, the conditions in which they are raised could have a strong impact on their fitness upon release or reintroduction. For example, lake substrate colour may induce a plastic response in wild hatchling turtles, potentially increasing their ability to camouflage and avoid predation (Cooper and Greenberg 1992). In this case, conservation managers could try and closely replicate these conditions in captivity to avoid inducing a maladaptive plastic response. In addition to morphological changes, there may be behaviours learned early in life, for example, relating to predator recognition, social and reproductive behaviour and obtaining necessary food and nutrients. To help ensure the success of these programmes, we should identify traits that exhibit developmental plasticity, the degree to which the plastic change is reversible or able to be exhibited later in life, and their fitness consequences in the release environment (Figure 4).

Beyond individual developmental plasticity, captivity may induce epigenetic changes that influence traits across multiple generations (Baker et al. 2019; Evans et al. 2014). These effects could persist even in offspring not directly exposed to the captive environment, potentially shaping phenotypes in ways that impact fitness post‐release. This highlights the need to understand how epigenetically mediated responses might alter phenotypes in the wild. Multi‐generational studies are essential to identify the extent and persistence of these effects, and to better understand their importance in the context of conservation releases and fitness surfaces.

### 3.4 Heterogeneity Among Facilities

Different facility environments and the transfer of animals among facilities could alter the fitness surface and result in plastic responses. In the simplest scenario, individuals experience one environment while being reared at a conservation breeding facility, and a different environment when introduced into the wild. However, individuals are often moved between breeding facilities to minimise inbreeding and maximise genetic diversity by pairing individuals based on minimising mean kinship (Lacy 1995). To be able to predict which individuals or genotypes will have the highest fitness post‐release, we not only need to understand how fitness surfaces differ in the wild and captive environments, but also how individuals and genotypes plastically respond to different captive environments and if and how fitness surfaces might vary among facilities.

### 3.5 Using Fitness Surfaces to Predict the Expected Average Fitness of Released Phenotypes

Our understanding of the wild fitness surface and the distribution of phenotypes we expect to be expressed in the release environment could allow us to estimate the expected mean fitness of the released individuals. Given the fitness surface and predictions for the released phenotypes, managers could calculate average fitness for the distribution of released phenotypes (e.g., estimating part of the adaptive landscape). Because the mean fitness of the released individuals is a function of the distribution of released phenotypes, it would be possible to explore the expected fitness consequences of a variety of distributions of released individuals (Arnold 2003; Lande 1976). Managers could try to balance a high expected average fitness for the released phenotypes with a diversity of phenotypes within the captive breeding programme. If the demographics of the wild population are well studied, managers might have a target average fitness they would like to achieve for the released phenotypes.

## 4 What Information Is Required Before We Can Use This Framework?

Before integrating fitness surfaces into conservation breeding programmes, there are a few data collection needs to address (Box 1; Figure 4). Currently, many breeding programmes maintain pedigrees, collect fitness data (e.g., reproductive success), and information on basic life‐history traits (e.g., egg laying date; Box 1; Figure 4). Further, much of this data are available to breeding partners and zoo researchers through international databases like ZIMS (ZIMS 2023). Ultimately, the amount of data required from the captive and wild environments will depend on whether phenotypic (co)variances align with additive genetic (co)variances (Section 3.1) and among individual and additive genetic (co)variation in the individual and genetic responses to the wild environment upon release (Section 3.2).

Beyond pedigree and fitness data, we need to answer the practical question: what traits should we be measuring? In many circumstances, this will require an understanding of the ecology, life‐history and changing environments of the species we are reintroducing (Box 1). However, global change literature and previous studies of traits in captivity have highlighted photoperiodic, disease, reproductive/sexual display and stress‐related traits as being particularly important (McPhee and Carlstead 2010; Parmesan and Yohe 2003; Schulte‐Hostedde and Mastromonaco 2015). Further, it seems logical that changes in parental care and predator avoidance behaviours would be detrimental to reintroduction success (de Bruijn et al. 2020; Gilby et al. 2011; Savage et al. 1996).

Understanding which traits are under selection and identifying the agents driving this selection is a critical next step. Comparative analyses of fitness surfaces between wild and captive populations, as well as the experimental release of specific phenotypes, could help identify selective pressures (Wade and Kalisz 1990). Ideally, these approaches can point to the traits most important for survival and reproduction in the wild. Questions of trait‐fitness causality are central to these efforts (e.g., Svensson 2023) because they emphasise the importance of distinguishing correlational associations from causal links when interpreting fitness effects. In the context of a conservation programme (whether wild monitoring or conservation breeding) understanding the ecological or anthropogenic agents of selection will be an important component of developing management actions.

Finally, in our view, molecular genetic and genomic work has the potential to fill in a lot of data gaps across the wild–captive continuum (Box 1). It can improve our estimates of relatedness from pedigrees, allow us to determine the relationship between wild and captive individuals, estimate relatedness among individuals in the wild, and potentially offer approaches to approximate fitness measures in natural environments (Gienapp et al. 2017; Rousset 2002). Further, in the future, molecular genetic and genomic approaches could be used to generate fitness surfaces for molecular genetic variation. So far, molecular fitness surfaces have been generated for genotypes at a particular locus, but measuring the dynamic genotype‐fitness relationship in both the wild and in captivity could improve our ability to make predictions about the expected fitness of a released individual based on genetic information (Blanco et al. 2019; de Visser et al. 2018).

BOX 1 **Suggested Steps for Incorporating Fitness Surfaces Into New or Existing Conservation Breeding Programmes.**

1. Assess capacity to collect data in and ex situ. For example, is there already an existing monitoring programme in place? What data are being collected? (Section 4)
2. Plan data collection (Section 4)
  - Phenotypic traits: choose based on what is known about the species' ecology. For example—what traits are thought to be important for survival, reproduction, and/or development? Are there any traits of concern that have been noted as potentially having diverged in the captive population compared to the wild population?
  - Fitness: assess multiple measures where possible. For example—survival, annual and lifetime reproductive success.
  - Environmental variables: choose based on what is known about the species' ecology. For example—are there any environmental variables thought to trigger important developmental or behavioural traits? Are there environmental variables thought to have strong impacts on fitness?
  - Relatedness among individuals—ideal to include if the data are available but can still use fitness surfaces without it. Relatedness can be calculated from pedigrees and/or molecular data (see point 7 of this box).
3. Data collection: collect the same phenotypic, fitness, environmental and relatedness data from captive and wild populations.

4 Generate fitness surfaces to evaluate (Section 1)
  - Phenotypic distribution of traits in situ and ex situ
  - What phenotypes have the highest predicted fitness in the release environment?
  - Do predicted fitness optima differ between wild and conservation breeding environments? Among different in situ environments (either geographically and/or temporally)? Among different ex situ environments (either different breeding facilities and/or among years?)
5 Choose individuals for release based on predicted fitness optima in release environment (Section 2.2)
6 Collect phenotypic, fitness, environmental, and relatedness data post‐release to evaluate: (Section 3)
  - Do individuals respond plastically to the new environment?
  - How does this impact fitness post‐release?
  - Are there differences among environments (in situ vs. ex situ, among in situ environments, among ex situ environments, across different years)
7 If relatedness data are available for multiple generations: (Section 2.1)
  - Evaluate heritability of traits
  - Do we see or expect to see evolution in traits in a given environment? (In and/or ex situ)
  - Assess evolvability of traits
8 Use information generated to inform programme management in future years, considering, for example:
  - Which traits have the largest predicted impact on fitness, and does this change among environments? (Section 3.5)
  - Are there particular traits that should be prioritised? For example, those that are likely to evolve or explain variation in fitness (Section 2)
  - Is there evidence of IxE or GxE in any of the traits measured? (Section 3)
  - Are there particular phenotypes or genotypes that should be prioritised for breeding and/or release? (Section 2)

*Note:* In small populations, it may not be possible to collect sufficiently large datasets for some analyses (e.g., animal models or selection gradients). However, even partial datasets can still be used to generate informative fitness surfaces. Over time, programmes will be able to accumulate sufficient data to support more detailed analyses and increase the precision of predictions.

## 5 Conclusions and Next Steps

In conclusion, integrating fitness surfaces into conservation breeding programmes could improve the success of reintroductions. By characterising fitness surfaces across captive and wild environments, managers can make more informed decisions about which individuals or genotypes to release and how to manage captive populations. While generating these surfaces requires data on pedigrees, fitness and traits, many captive and wild conservation programmes already collect this information, providing a foundation for implementation. Molecular and genomic tools can further fill in data gaps and enhance predictive accuracy. Despite the challenges in forecasting evolutionary responses due to environmental variation and complex selection dynamics, long‐term investment in this approach may offer benefits (Box 1). Fitness surfaces have the potential to ensure captive populations and recovery actions are better integrated and support the recovery of wild populations, ultimately providing a better ecological and evolutionary understanding of managed populations (Box 1).

## Author Contributions

D.S. and H.T. formulated the idea for the paper. D.S. and H.T. wrote the original draft and produced all figures. D.S., H.T., D.R. and A.A.C. all commented on and revised the manuscript. D.S., D.R. and A.A.C. helped acquire funding for the research.

## Disclosure

Benefits generated: Benefits from this research will accrue from sharing ideas and results in this manuscript with conservation practitioners to determine where quantitative genetic techniques and measuring selection in endangered populations might benefit their management.

## Conflicts of Interest

The authors declare no conflicts of interest.

## Data Availability

No data were generated or analyzed as part of this review.
